# Extending a COVID-19 Job Exposure Matrix: The SARS-CoV-2 or COVID-19 Job Exposure Matrix Module (SCoVJEM Module) for Population-Based Studies

**DOI:** 10.3390/ijerph22030448

**Published:** 2025-03-18

**Authors:** Ximena P. Vergara, Kathryn Gibb, David P. Bui, Elisabeth Gebreegziabher, Elon Ullman, Kyle Peerless

**Affiliations:** 1 Heluna Health, 3300 Crossroads Pkwy. N #450, City of Industry, CA 91746, USAelisabeth.gebreegziabher@cdph.ca.gov (E.G.);; 2 California Department of Public Health, Occupational Health Branch, 850 Marina Bay Pkwy. P-3, Richmond, CA 94804, USA; 3 Public Health Institute, 555 12th Street, Oakland, CA 94607, USA

**Keywords:** COVID-19, aerosols, job exposure matrix, occupational exposure assessment

## Abstract

The risk of workplace SARS-CoV-2 transmission is increased by aerosolization or droplets and increased respiratory rates or increased viral stability in cold environments. Few methods exist for identifying occupational risks of SARS-CoV-2 transmission. We extended a SARS-CoV-2 job exposure matrix (JEM) into four dimensions, talking loudly (Loud) (very loud, loud, somewhat loud, or not), physical activity (PA) (high, medium or low), and cold (Cold) (cold or not) and hot environments (Hot) (hot or not), using data from the Occupational Information Network (O*NET) and a priori questions for each and noise measurements for 535 occupations. We classified 70%+ occupations as loud or very loud (74.6%); whereas 13.8% were high PA, 18.5% exposed to cold, and 23.7% exposed to hot temperatures. Applying to California 2019 workforce data to explore by race/ethnicity and sex, we found 21.2% worked in very loud and 12.6% in high PA occupations and 15.7% in cold and 17.8% hot environments. Latino workers were highly represented in very loud and high PA levels among farming (83.8 and 78.4%) and construction (58.7% and 50.3%). More males worked in each highest exposure level than females. This JEM provides aerosol transmission proxies for COVID-19 risk factors and merits investigation as a tool for epidemiologic studies.

## 1. Introduction

Data describing the extent of workplace SARS-CoV-2 exposure and occupationally acquired COVID-19 illness remains fractured within a patchwork of studies. California public health researchers have described outbreaks occurring within manufacturing and healthcare sectors [1], and excess and cause-specific mortality among agricultural, transportation, material movers, and protective service occupation groups [2,3]. Occupation alone, however, serves as a proxy for workplace exposures that might increase the risk of SARS-CoV-2 infection. Moreover, recent population attributable fraction approaches estimate one in four infections are due to work [4,5]. Consequently, delving deeper than occupation is necessary for elucidating specific risk factors for COVID-19.

The exploration of workplace risk factors for COVID-19 remains in its nascent phases, primarily owing to a lack of exposure assessment methods. Job exposure matrices (JEMs) cross classify occupations by exposure dimensions [6]. In 2022, an International Standard Classification of Occupation-based JEM for COVID-19 was developed using data from three European countries. This European JEM considers the number and nature of contacts, social distancing, masking, contaminated workspaces, location, income insecurity, and migrant status [7]. In another study, researchers developed a JEM with the probability of contact with people, time, and location factors associated with circulating virus and prevention methods for use within the French worker population [8]. Each of these JEMs illustrates an exposure assessment approach based upon infectious disease principles but not an industrial hygiene approach. US-led efforts to assess the types of contacts, physical proximity, and indoor environment among non-healthcare workers resulted in a JEM for US-based occupational codes known as the SARS-CoV-2 Occupational Exposure Matrix (SOEM) [9,10]. When combined with SARS-CoV-2 exposure and infection data containing coded occupations, JEMs can assist in characterizing worker populations at risk of COVID-19 and disentangle potential risk factors. Since the SOEM considered three dimensions of exposure for the working US population, expansion of other potential exposure risk factors addressing aerosol transmission is needed.

Scientists recognized the importance of aerosol transmission of SARS-CoV-2 based on super spreader events, e.g., choral practice [11]. Worker activities or worksite characteristics may affect the generation and transmission of aerosols including activities involving frequent and sustained physical activity (PA), resulting in higher respiration rates, or loud environments, which may require greater vocal effort and result in greater respiratory aerosol emission [12,13]. Temperature affects respiratory susceptibility and aerosol behavior. Low temperatures can keep viral-laden particles environmentally stable for longer, hence, we might consider whether someone might work in a cold environment [14,15]. In contrast, hot work environments may not provide an environmentally stable environment for SARS-CoV-2 particles. For exposure to SARS-CoV-2 or any other airborne viral infections [16], we expanded a COVID-19 JEM combining existing data and judgment to identify occupations within which workers need to speak loudly, exert PA, conduct activities in cold environments, or perform duties in hot environments. In addition, we assessed the number of workers in California potentially classified as needing to speak loudly, exert high levels of PA, and perform duties in cold and hot environments to identify potentially at risk workers. Our work satisfies several objectives to (1) document an occupational exposure assessment approach for several potential risk factors for SARS-CoV-2 exposure, (2) identify occupations with the highest proportions of California workers with these risk factors, (3) assess the racial/ethnic distribution of workers with these risk factors, and (4) compare our approach to a subgroup of occupations to other US survey data. These tools can assist public health practitioners in assessing their own worker populations.

## 2. Materials and Methods

To extend the SOEM JEM, we used existing data on the nature of the workplace through the Bureau of Labor Statistics Occupational Information Network (O*NET) program [17]. O*NET maintains a database with descriptive information on the workplace characteristics, knowledge, skills, and abilities of detailed occupations in the US. O*NET data are derived from worker surveys of each occupation and input from occupational health experts. O*NET surveys are collected on occupations from a predetermined taxonomy and updated quarterly. O*NET is designed to create and update the O*NET database in a highly cost-efficient and timely manner by sampling as a two-stage collection process: first, at the establishment level and, second, a sample of employees is selected within certain occupations within an establishment. Employees are asked to complete one of the four O*NET domain questionnaires. O*NET surveys for each occupation are published if there are at least 15 respondents for the four domains.

As of December 2017, O*NET surveyed 180,153 establishments and 213,603 employees with response rates of 75% and 64%, respectively, since 2011 [18]. Industry analyses of the O*NET data using the SOEM focused on non-healthcare working occupation exposures, like contact with others, physical proximity, or indoor/outdoor. These analyses found over 80% of the US workforce were employed in high risk occupations dominated by accommodation and food service, social assistance, healthcare, and educational services [19].

Talking loudly, PA, and cold and hot temperatures were the workplace exposure dimensions we assessed in O*NET v25.0 to create the SCoVJEM module.

We implemented a general approach to developing each exposure dimension with the aim of creating an extension of the SOEM JEM, based on US Bureau of Census Occupation Codes (COC 2010). For each exposure dimension, we conducted a literature search to understand how it is measured, developed an a priori list of questions used to assess each measure using our own knowledge informed by published literature, and performed manual assessments for each of the 968 O*NET Standardized Occupation Classification (SOC) occupations. In general, the average sample size per O*NET SOC occupation for each question was 27 respondents, except in the case level of stamina and explosive strength, which were smaller. We integrated additional information such as measurement data and/or author review (epidemiologists and industrial hygienists), explored O*NET for suitable information, and combined all information into one measure. We also tailored specific methods for developing each exposure measure to accommodate differences in exposure domains, number of exposure tiers, and data availability as outlined below.

### 2.1. Dimensions

#### 2.1.1. Talking Loudly

For talking loudly, we created three distinct measures which incorporate different data sources: (1) using O*NET data alone, (2) using O*NET data and secondary data on average occupational noise exposures, and (3) using judgment.

First, we conducted a literature search on noise-induced hearing loss and loud environments. Next, we summarized potential risk factors for risk of being in a loud environment or noise-induced hearing loss and devised a three a priori question framework to assess each of the 968 O*NET SOC codes (Table S1). Two authors (XV and KG) reviewed all codes, independently assessing which occupations were anticipated to work in very loud environments (yes/no) by asking whether the occupation involved any of the listed criteria (Table S1).

O*NET questions used to construct the talking loudly exposure measure were the frequency of working in loud environments, dealing with physically aggressive people, and having face to face discussions (Table S1). We reviewed score distributions, correlations, and set thresholds based on percentiles correlated to five-point responses for O*NET question scores to create a composite measure for talking loudly. For the O*NET composite measure (Loud O*NET), the four exposure levels were not loud, somewhat loud, loud, and very loud. For example, a worker in the loud occupation would encounter loud situations at least weekly and face to face discussions at least weekly, but not daily; or less than weekly for loud or face to face discussions but dealing with aggressive people monthly. Whereas a worker in a somewhat loud occupation would be encountering loud situations monthly, but not weekly and dealing with aggressive people less than monthly.

To create a second exposure measure for talking loudly (Loud O*NET + Measurement (Meas)), we combined publicly available measurement data compiled by the University of Michigan NoiseJEM with the Loud O*NET scores [20]. The NoiseJEM data included 8-hour, time-weighted, average noise exposures by occupation (using broad level SOC Codes) available from 1963 to 2015. We used Occupational Safety and Health Administration (OSHA) permissible exposure limit (PEL) estimates from 2000 onward and created exposure levels informed by the OSHA action level (85 A-scale decibels (dBA)). The exposure levels were not loud (≤69 dBA), somewhat loud (70–79 dBA), loud (80–84 dBA), and very loud (≥85 dBA). We applied the exposure levels from broad SOC codes to nested SOC codes to create detailed SOC measurement data. If the noise measurement exposure level was higher than Loud O*NET, we increased the exposure level assignment, resulting in 86 O*NET SOC exposure level changes from a lower exposure level to very loud (Loud O*NET + Meas).

To create our third measure for talking loudly (Loud O*NET + Meas + Judgment (Judg)), we integrated the a priori manual review of the O*NET SOC codes, resulting in 71 O*NET SOC codes changing from a lower exposure level to very loud.

#### 2.1.2. Physical Activity

Based on a literature review, we constructed an a priori five question rubric to assess occupational PA (Table S1). Three authors (D.B., E.G., X.V.) independently used the rubric to assess and rank all 968 O*NET-SOC occupations into low, medium, or high PA levels (PA O*NET + Judg).

We then constructed a PA score using four O*NET ratings of the importance and level of stamina, explosive strength, general PA, and climbing indicated for each occupation (Table S1).

O*NET scores for each of the four PA domains were standardized according to the Brookings method [21]; for domains with an importance score and level score (stamina, explosive strength, and general PA), we summarized the score by taking the geometric mean of the two measures. The final four standardized scores were summarized by taking their arithmetic mean to arrive at a continuous summary measure of PA for each occupation with higher scores indicating higher PA (PA O*NET). To translate this continuous measure into categories of low, medium, and high PA, we selected cutoffs for each category to match the distribution of occupations in each PA level with those from our a priori assessment.

The team convened to review initial ranks and resolve disagreements. After discussion, the team agreed on a PA level for 933 (96%) O*NET SOC codes and sent the unresolved 35 (4%) occupations to two authors (KP, EU) to independently review and rank using the same rubric. Once the two authors completed their assessments, the team reconvened to discuss their rankings and resolved most of the disagreements (23, 66%) through consensus.

Next, we used an algorithm to adjust our a priori PA rankings to account for disagreements with the O*NET rankings. For any disagreement (*n* = 227), we took the three authors’ initial rank if the team had a unanimous initial ranking (*n* = 46). For the remaining 1-degree disagreements (e.g., low vs. medium or medium vs. high), we used the team’s initial rank if there was an initial majority (*n* = 144) and the PA O*NET rank if otherwise (*n* = 18). For the remaining (*n* = 19) two-degree disagreements (i.e., low vs. high), we manually reviewed each disagreement and resolved them through consensus (PA O*NET + Judg). After our a priori assessment, 12% of occupations were considered high PA, 20% were medium, and the remaining 68% were low; we selected cutoff points for the O*NET score to match this distribution.

#### 2.1.3. Cold and Hot Environments

We first conducted a literature review to identify known heat- or cold-exposed occupations or information about workplace temperatures. Using information from the literature, we devised a multi-question rubric to ask, “Does this occupation involve?” (Table S1). In general, we focused only on the occupational environment rather than exposure modifiers, e.g., personal protection equipment (PPE) on the job such as fire-retardant gear.

To construct cold and hot dimensions, a single question on working in extreme temperatures was used from O*NET (Table S1). O*NET standardized scores (70 or higher) were categorized as “exposed to hot or cold”, as a single measure (Extreme O*NET) and were later separated into two dimensions referred to as Cold O*NET and Hot O*NET.

We then developed two separate, binary measures for cold and hot using professional judgment alone guided by a set of a priori questions (Cold O*NET + Judg and Hot O*NET + Judg). Industrial hygiene (IH) reviewers consulted other online resources describing the tasks carried out or equipment used by specific occupation titles for which we were less familiar.

We combined the a priori exposure levels to assign whether an occupation might be working in very cold environments or very hot environments by assessing our initial hygiene review and the O*NET scores. After which, we resolved disagreements and reassessed the assignments for occupations we were less certain. Using the O*NET standardized score alone, we initially disagreed on 136 (14%) O*NET SOC codes and classified 85 (8.7%) occupations as exposed to cold, 94 (9.8%) occupations as exposed to hot environments, and 102 (10.5%) occupations as exposed to either hot or cold environments, of which 77 occupations were exposed to both. After our a priori assignment, 16.7% of the 968 occupations were considered exposed to cold and 20.2% of the occupations were considered exposed to hot environments.

Once dimensions were developed at O*NET SOC level, we converted them first into 2010 SOC, then into 2010 COC using previously described methods [22].

#### 2.1.4. Index

To combine all dimensions, we assigned an occupation to a high level if it was very loud, had high PA, and was exposed to cold but not hot environments. The occupation was assigned to the medium level if two of the preceding dimension criteria were met. Otherwise, we designated the occupation at the low level.

### 2.2. Workforce Estimates

California workforce 2019 estimates (coded to 2010 COC) were extracted from the National Institute for Occupational Safety and Health Employed Labor Force Query system and were used to derive estimates for each JEM exposure level [23].

Occupational estimates by racial/Latino ethnicity (Latino, White, Asian, Black, Hawaiian/Pacific Islander, American Indian Alaskan Native [AIAN], and Multirace) and sex were also retrieved. There were 81 occupations without racial/Latino ethnicity subgroup estimates. We analyzed data by major occupational groups both as percentages of occupations and of California workforce estimates to examine the potential for occupational exposure to be very loud, strenuous physical activity, and cold and hot work environments.

### 2.3. Comparison to 2018 Occupational Requirements Survey

As validation data for these potential risk factors do not exist, we sought to compare and obtained 2018 Occupational Requirements Survey (ORS) Estimates from wave 1 (N = 14,080,000) from 25,300 establishments coded to O*NET-SOC 2010 occupations. After which, we created four new binary dimensions from several variables from the ORS to mimic the SCOVJEM dimensions. ORS provides dimensions as percent distribution across an occupation, for example, loud were percentages across low, moderate, and high intensities. For talking loudly, we classified workers as exposed if 5% of workers worked in loud noise intensity level, with a low threshold to capture any level of loud intensity. For physical activity, if any of these variables met the indicated percentages or criteria, we classified them as high physical activity: climbing ladders, ropes, or scaffolds (5%), climbing ramps or stairs (structure- or work-related) (50%), using strength for heavy or very heavy work, or lifting/carrying heavy items (10 lbs or more) frequently. For extreme heat and extreme cold, we considered workers exposed if the percentage of workers with no exposure was less than 90%. If O*NET SOC occupations needed to be collapsed into a single SOC, we averaged the binary variable. After which we converted these data to COC. To create comparison groups for the SCoVJEM loud category, we combined very loud and loud into the exposed group. We then compared ORS-derived frequencies to the SCOVJEM using kappa statistics (poor (<0.40), moderate (0.41–0.75), substantial (>0.75)), Sensitivity (Se), and Specificity (Sp).

## 3. Results

### 3.1. Overall Description

We created an extended exposure matrix for aerosol transmission, talking loudly (four-level exposures), PA (three-level), cold (two-level) and hot (two-level), resulting in 535 COC-based occupations. Overall, we classified over 70% or more occupations as loud or very loud (74.6%); whereas 13.8% were high PA, 18.5% were exposed to cold, and 23.7% were exposed to hot temperatures (Table 1). Moreover, 35 occupations were assigned to the highest exposure categories for all four dimensions, among which 21 (60%) were construction and extraction (construction). When applied to the California workforce 2019 estimates, we found 21.2% worked in very loud occupations, 12.6% worked in high PA occupations, and 15.7% were exposed to cold and 17.8% to hot temperatures.

#### 3.1.1. By Occupation

**Figure 1:**
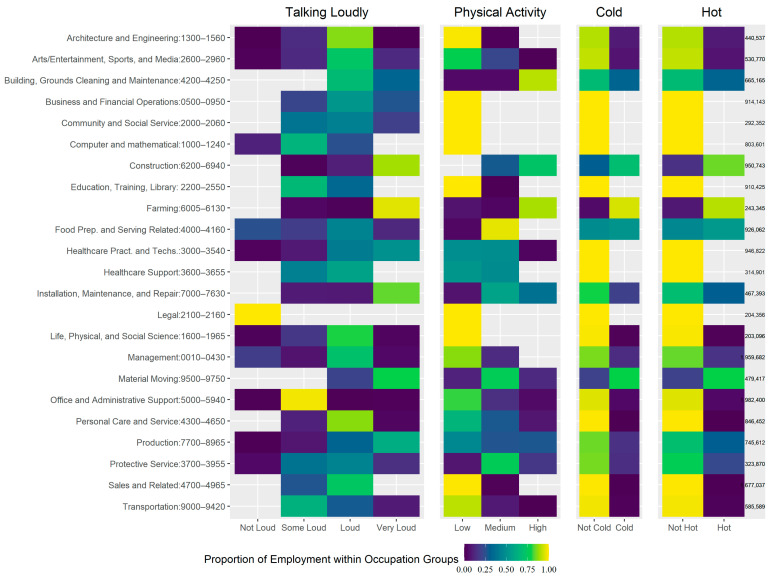
Proportion of California workers by occupation group within level of exposure dimension using SARS-CoV-2/COVID job exposure matrix module. Notes: Using Current Population Survey estimates for California, 2019; Construction: Construction and Extraction; Farming: Farming, Forestry, Fishing; Healthcare Pract. and Techs: Healthcare Practitioners and Technicians; Arts, Design, Entertainment, Sports, and Media: Arts/Entertainment, Sports, and Media.

The most frequent major occupation groups in the very loud category were the construction, material moving, production, and farming, fishing and forestry (farming) groups (Table 2). Occupational groups with the highest proportion of workers in the very loud category were farming (95.4%), construction (89.0%), installation, maintenance and repair (83.2%), and material moving (79.0%) (Figure 1).

The most physically active occupations were concentrated in the construction and farming groups (Table 1). Occupational groups with the highest proportion of workers in the high PA category were building, grounds cleaning and maintenance (91.3%), farming (89.7%), construction (72.0%), installation, maintenance and repair (36.7%), and production (27.1%) (Figure 1).

Cold and hot work environments mainly involved occupations among the farming, material moving, and construction groups (Table 1). Occupational groups with the highest proportions of workers in the cold and hot environment category, respectively, were farming (94.3% and 91.1%), material moving (79.4% and 79.4%), construction (69.4% and 84.5%), food preparation- and serving-related (51.9% and 53.9%), building, grounds cleaning and maintenance (32.4% and 32.4%), installation, maintenance and repair (20.2% and 30.6%), and production (15.6% and 30.3%) (Figure 1).

#### 3.1.2. By Race/Latino Ethnicity

For each racial/Latino ethnicity group, we found at least 15% of the workers were categorized as working in very loud occupations. Within racial/ethnic groups, over 15% of AIAN and Latinos worked in high PA occupations, and over 10% of each racial/ethnic group except Asians worked in cold and hot environments. Among all Latino workers, 28.1% worked in very loud occupations, 22.1% were high PA workers, and 24.6% worked in cold and 27.5% in hot environments. AIAN were also among those working in very loud occupations (26.7%), carrying out high PA work (15.0%) and working in cold (21.6%) and hot (21.6%) environments. Within occupation groups, Latinos comprised the highest proportion of the workers in the very loud and high physical activity categories among farming (83.8% and 78.4%), construction (58.7% and 50.3%), material moving (50.2% and 9.8%), and installation, maintenance and repair groups (42.6% and 18.9%). Latino workers represented over half of the workforce within three of the preceding occupational groups: farming (87.0%), building, grounds cleaning and maintenance (75.2%), and construction (64.4%). Similarly to patterns observed for workforce trends in occupation groups for very loud and PA, Latinos represented over 40% of the workforce exposed to cold environments in the material moving and construction groups and over 80% in farming (Table 2).

#### 3.1.3. By Sex

Among all workers, more males worked in very loud environments (difference: +10.4%), high PA occupations (+7.2%), cold environments (+9.1%), and hot environments (+10.4%) compared with females. Males in very loud occupations were mainly in the construction (86.7%), installation, maintenance and repair (81.0%), farming (66.2%), and material moving groups (65.7%); whereas females were mainly in healthcare practitioners (37.6%) and farming (29.1%). Over 50% of the high PA occupations among males were in construction (70.2%), farming (64.6%), and building, grounds cleaning and maintenance groups (53.4%). Most high PA occupations among females were in two occupational groups, building, grounds cleaning and maintenance (37.9%) and farming (25.0%). Male workers exposed to cold environments were within the farming (68.4%), construction (68.0%), and material moving groups (66.3%). Occupational groups with the highest proportion of women exposed to cold environments were the farming (25.9%) and food preparation- and serving-related groups (24.2%). The patterns for hot environments were somewhat similar to cold for both sexes.

#### 3.1.4. By Index

Three occupations were assigned to a high exposure level by the index, comprising a small proportion of the workforce (0.1%). These were fishers and related fishing workers (COC: 6100), butchers and other meat, poultry and fish processing workers (7810), and sailors and marine oilers (9300). Occupations assigned to the medium exposure level could work both in cold and heat. Of the 57 occupations in the medium exposure level, many were among workers in construction (45.6%), installation maintenance and repair (19.3%), and production (12.3%). Overall, most of the workforce (91.7%) was categorized as the low exposure level by the index.

### 3.2. Comparison

**Figure 2:**
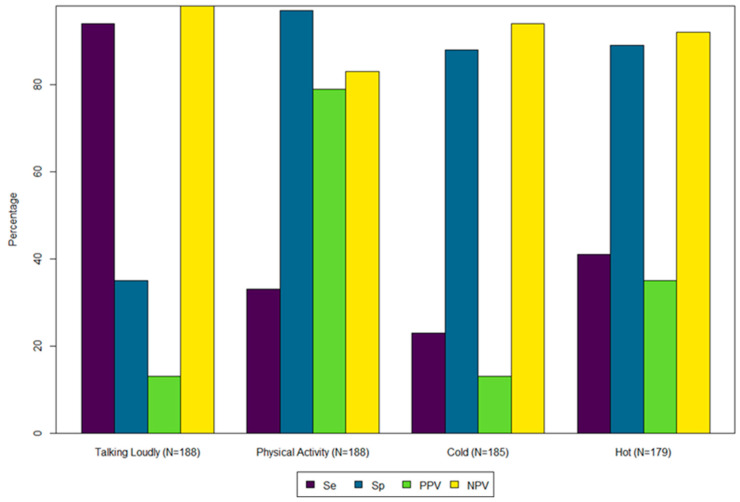
Comparison (Sensitivity, Specificity, Positive Predictive Value, and Negative Predictive Value) of Occupational Requirement Survey data to SARS-CoV-2/COVID job exposure matrix module. Notes: Se = Sensitivity; Sp = Specificity; PPV = Positive Predictive Value; NPV = Negative Predictive Value.

For the 188 occupations we could assign exposures using ORS, we found poor agreement for loud (kappa = 0.0184) and physical activity (kappa = 0.3807). While for the 179 occupations for cold and heat, we also found poor agreement for each (kappa = 0.0781 and kappa = 0.2790, respectively). High Se (>0.80) was obtained for talking loud and high Sp (>0.80) was obtained for high physical activity and cold and hot environments (Figure 2).

## 4. Discussion

We created a JEM extension, the SCoVJEM module, to define occupations at risk of exposure through aerosol transmission focusing on which workers need to speak loudly, exert PA, or conduct activities in cold and hot environments, which is critical to disentangle risk factors for SARS-CoV-2 or other airborne respiratory viruses within epidemiologic studies. To our knowledge, our developed tools are a first attempt to examine these particular risk factors for SARS-CoV-2 exposure. We found that over one-fifth of the California workforce worked in very loud environments and over a tenth worked in high PA jobs or in cold and hot environment jobs based on O*NET and other information.

Early on during the pandemic, scientists recognized that talking loudly within densely populated and poorly ventilated environments would increase transmission risk [24]. Tak et al. estimated that 17% of US workers were exposed to noise on the job [25]. Our estimate for workers employed in very loud occupations was similar. Zhang found occupational disease and physical proximity using O*NET alone predicted Washington state (WA) COVID-19 incidence; in response, Shkembi and Neitzel found a 33–57% higher incidence rate of COVID-19 associated with a 3 dB increase in occupational noise with the WA data [26,27]. Noise remains an underappreciated workplace exposure for a variety of outcomes including COVID-19 [28].

Our JEM coupled with population data estimate that many Californians work in very loud occupations and in high PA jobs. Vocalization and physical intensity were factored into newly developed models by Peng et al. for understanding COVID-19 outbreaks [29]. These models suggest the most benefit from strategies when used together, for example, reducing vocalization and avoiding intense physical activities. However, these strategies may not be possible in a noisy work environment or when workers use large muscle groups for sustained periods of time for physically demanding jobs. Moreover, workers in noisy environments may need to work closer together to be able to hear one another. This SCoVJEM, when coupled with COVID-19 data or with studies evaluating mitigation measures, may assist to disentangle why particular occupations might be at higher risk.

Lower temperature and humidity were observed to be associated with COVID-19 risk, possibly due to viral persistence [30]. Temperature can also affect the infectivity decay rate of the virus as an aerosol and is included in the models of COVID-19 outbreaks [29]. If the workers who are exposed only to cold have a higher risk of COVID-19 infection versus those exposed to both cold and hot, then it would lend credence to the viral persistence hypothesis. Cold temperatures may place workers in farming, material moving, and construction at increased risk for COVID-19 infections, which can be explored using the SCoVJEM coupled with COVID-19 data containing occupation.

When we examined California workers, more than 50% of workers in the farming and construction groups were the highest exposure level in all dimensions of the SCoVJEM. These occupational groups bore a high burden of COVID-19 mortality [3], yet the reasons for which are unexamined except in a few studies. Despite that SCoVJEM does not account for either workplace or personal protective measures, we must consider how these risk factors interplay within work environments using the literature. In agriculture, maintaining physical distances of six feet or greater was reported as challenging at times [31]. Close physical proximity combined with loud work environments might increase the transmission of SARS-CoV-2, for example. Improved ventilation is often recommended as the highest tier IH control measure for aerosol transmission, but may be infeasible in some workplaces, e.g., indoor construction sites with uninstalled or inoperable heating ventilation and air conditioning systems [30]. For studies that have collected information about the types of ventilation and detailed occupation, an assessment of proximity and/or loud environments stratified by types of ventilation might be possible [32]. A 50% reduction in transmission can be achieved when mitigation measures, such a masking, and limiting worker density, as supported by models of worksite infections among construction, are implemented [33]. Masks and respirators, widely recommended to interrupt aerosol transmission during the pandemic, however, may not have been used in very loud work environments where workers with hearing loss rely on non-verbal cues or lip-reading [34,35]. Nonetheless, workplace COVID-19 epidemiology requires in-depth discussion supported by findings from mitigation strategy evaluation studies using JEM to understand and interpret epidemiologic findings overall. Six states, including California, collected surveys during the pandemic on test-positive workers and mitigation measures [36]. California used the SOEM and found increased odds of test positivity among those who worked in very close physical proximity. These data might also be used with SCoVJEM [37]. This SCoVJEM, when coupled with other COVID-19 data containing both occupation and workplace-level strategies, may generate insights.

This SCoVJEM module uses potential workplace factors suspected to place workers at risk for SARS-CoV-2 that are different from other studies previously published. Occupations we identified using the index of exposure differ somewhat from those listed by the Occupational Safety and Health Administration (OSHA) as very high to high risk for SARS-CoV-2 exposure (registered nurses, personal care aides, physicians, and surgeons) [38]. This is not unexpected given that OSHA’s determination was based on the potential for encountering those infected with SARS-CoV-2. Our SCoVJEM module assesses occupations by other potential risk factors but finds overlap with others assumed to be in the medium OSHA category due to working in densely populated environments (firefighters, construction laborers, and cleaners of vehicles and equipment) or high-volume retail settings [38].

California researchers assessed the racial/ethnic composition of the 2018 California workforce, finding overrepresentation for Latino workers in agriculture, construction, trucking, material moving and stocking, and cooks and food preparation, Asian workers in registered nurses and personal care aides, Black workers in personal care aides, laborers and material movers, and food preparation workers, and White workers in secretaries, supervisors of retail workers, and registered nurses [39]. Identifying these populations as high risk is consistent with the prior literature on COVID-19 mortality [2,3]. Reitsma et al. found that 44.8% of California populations living in households with an essential worker were Latinos, followed by Whites (31.7%), Asians (15.4%), and Blacks (4.5%) [40]. We described racial/Latino ethnicity composition and exposure level together within occupation groups and our findings show non-White racial/ethnic workers were among our highest exposed for talking loudly, high PA, and cold and hot parameters. However, these characterizations were not stratified by other important factors such as age and sex. Regardless, statewide characterization of the workforce lays the foundation for the interpretation of epidemiologic studies of occupation and COVID-19.

Our assessment of the SCOVJEM module compared to external data yielded poor agreement, but high Se for talking loudly and high Sp for physical activity and hot environments. However, this comparison was limited by the fact that only ~35% of occupations could be compared due to a lack of data available within the ORS set. These occupations were mainly in the office and administrative support, business and finance, management, production, and healthcare practitioners and technical groups. A bias towards higher wage earners or higher educated workers might be indicated. More importantly, workers who may not be exposed to loud environments or temperature extremes. Handel examined O*NET survey data in relation to wages, finding negative correlations with several of the O*NET questions we employed [41]. Together, this lends support for a degree of selection bias for both of these data sets. Another key difference between our comparisons with ORS is that we combined measurement and judgment for the loud dimension and judgment with the cold dimension. Yet a gold standard does not exist and data for other comparisons are not readily available for this SCoVJEM module.

The application of the JEM module will include linkage to COVID-19 data sources containing occupational data such as electronic death certificates, survey data, occupational injury/illness reports, and workers’ compensation (WC) claims data. As a case example, we recently linked SCoVJEM to coded occupations within WC data and found that both very close physical proximity and high physical activity associated with being part of a large cluster of COVID-19 exposure or illness claims related in time and space (worksite). These findings suggest that layered mitigation strategies in a workplace are warranted for the prevention of aerosol transmissible respiratory viruses [42].

Several limitations are worth discussing. First, SCoVJEM relied on information with original O*NET questions and O*NET was not intended to be an exposure database for population research. As such, certain information that may have also accurately approximated exposure risk were either not available in ONET data or were not as straightforward to approximate using professional judgment as the factors studied. Furthermore, the surveys for certain jobs are based on a limited sample size—possibly reflecting industries and practices in a particular region. Even though O*NET augments with occupational experts, we also used our own judgment, which is limited by our knowledge of each occupation, and other external data sources to describe jobs with limited information on the tasks or environments encountered by a worker.

Second, limited measurement data for detailed occupations were available for the talk loudly dimension. In some cases, data collected to broad SOC codes were applied to all nested detailed occupation codes. This approach could result in an overestimation of exposure risk since we did not have reason for the measurement data in NoiseJEM. We overcame these limitations by incorporating our judgment for the occupations overall. On the other hand, survey data alone may underestimate loud work environments, especially if hearing decrements were present among respondents. Examples of low exposure based on the O*NET alone assignment were both bartenders (O*NET-SOC 35-3011.00) and slaughterers and meat packers (O*NET-SOC 51-3023.00), which, when combined with measurements and judgment, shifted into very loud exposure. Future work might compare COVID risk estimates derived from the talking loudly dimension using Loud O*NET and Loud O*NET + Meas + Judg.

There are several other significant limitations to the SCoVJEM module. First, we constructed four exposure dimensions across an occupation, which may be heterogeneous for these exposures. An occupation encompasses several different occupational titles and varies depending on the industry. Also, the literature characterizing population specific occupations exposed to cold work environments is scant. This dimension along with the hot work dimension can inform future occupational health and climate work. Lastly, this JEM was developed for the 2010 COC classification using a snapshot of occupational information. COC is broader than fine-level O*NET-SOC and SOC codes; hence, our grouping would dilute any heterogeneity and pull exposures toward an average; the direction of change would depend on the occupations within the data set and the exposure dimension. Hence, even the cross walked data may require additional assumptions about exposures.

We recognize that 2019 workforce estimates do not reflect the pandemic workforce. Pandemic-related job losses were unevenly distributed across sex and race/ethnicity categories, with women and people of color deeply impacted during the first year of the pandemic [43,44]. Moreover, our examination of the workforce does not account for other explanatory factors such as age, sex, or co-morbidities within a workforce.

Finally, occupation serves as a surrogate measure for a host of exposures; the factors placing workers at risk for SARS-CoV-2 exposure or which mitigation measures were most effective is yet unknown. In conjunction with other newly developed tools such as SOEM JEM, the SCoVJEM module builds on the current work in the occupational health literature. However, several risk factors not included in this JEM module could place a worker at risk of SARS-CoV-2 exposure in the workplace. These factors include the number and types of contacts encountered, the type of ventilation and whether work was conducted indoors, whether personal protective equipment was available and used, whether mitigation measures such as physical distancing were possible and used, among others. However, JEM as tool for evaluating the intersection of infectious diseases and climate change is valuable.

## 5. Conclusions

After several years of a global pandemic, we are emerging with new ways to examine the burden of COVID-19 on workers. The development of the SCOVJEM module is a way to assess whether workers may speak in elevated voices, perform high PA, and work in cold and/or hot environments using workforce estimates. These tools can also be coupled with data on COVID-19 outcomes to assess whether workplace factors are associated with exposures or illnesses. Combined with studies that have occupation information and examine the implementation of mitigation measures, we may be able to determine which interventions were more successful and target other public health measures towards specific occupations or workplaces.

## Figures and Tables

**Table 1:** Distribution of occupations by each dimension and level of SARS-COV-2/COVID-19 job exposure matrix module.

		Talking Loudly (O*NET + Meas + Judgment)				Physical Activity (O*NET + Judg)			Cold (O*NET + Judg)		Hot (O*NET + Judg)		Index		
Occupation Group: COC Range	N	Not Loud	Some Loud	Loud	Very Loud	Low	Medium	High	Not Cold	Cold	Not Hot	Hot	Low	Medium	High
Architecture and Engineering: 1300–1560	21	1 (4.8%)	4 (19.0%)	14 (66.7%)	2 (9.5%)	18 (85.7%)	3 (14.3%)	0 (0%)	17 (81.0%)	4 (19.0%)	17 (81.0%)	4 (19.0%)	21 (4.4%)	0 (0%)	0 (0%)
Arts/Entertainment, Sports, and Media: 2600–2960	19	1 (5.3%)	3 (15.8%)	13 (68.4%)	2 (10.5%)	13 (68.4%)	5 (26.3%)	1 (5.3%)	18 (94.7%)	1 (5.3%)	18 (94.7%)	1 (5.3%)	19 (4.0%)	0 (0%)	0 (0%)
Building, Grounds Cleaning and Maintenance: 4200–4250	6	0 (0%)	0 (0%)	4 (66.7%)	2 (33.3%)	1 (16.7%)	2 (33.3%)	3 (50.0%)	4 (66.7%)	2 (33.3%)	4 (66.7%)	2 (33.3%)	5 (1.1%)	1 (1.8%)	1 (1.8%)
Business and Financial Operations: 0500–0950	28	0 (0%)	6 (21.4%)	21 (75.0%)	1 (3.6%)	28 (100%)	0 (0%)	0 (0%)	28 (100%)	0 (0%)	28 (100%)	0 (0%)	28 (5.9%)	0 (0%)	0 (0%)
Community and Social Service: 2000–2060	8	0 (0%)	1 (12.5%)	4 (50.0%)	3 (37.5%)	8 (100%)	0 (0%)	0 (0%)	8 (100%)	0 (0%)	8 (100%)	0 (0%)	8 (1.7%)	0 (0%)	0 (0%)
Computer and mathematical: 1000–1240	16	1 (6.3%)	4 (25.0%)	11 (68.8%)	0 (0%)	16 (100%)	0 (0%)	0 (0%)	16 (100%)	0 (0%)	16 (100%)	0 (0%)	16 (3.4%)	0 (0%)	0 (0%)
Construction: 6200–6940	40	0 (0%)	1 (2.5%)	4 (10.0%)	35 (87.5%)	0 (0%)	11 (27.5%)	29 (72.5%)	10 (25.0%)	30 (75.0%)	8 (20.0%)	32 (80.0%)	14 (2.9%)	26 (45.6%)	25 (45.6%)
Education, Training, Library: 2200–2550	11	0 (0%)	10 (90.9%)	1 (9.1%)	0 (0%)	10 (90.9%)	1 (9.1%)	0 (0%)	11 (100%)	0 (0%)	11 (100%)	0 (0%)	11 (2.3%)	0 (0%)	0 (0%)
Farming: 6005–6130	9	0 (0%)	2 (22.2%)	1 (11.1%)	6 (66.7%)	2 (22.2%)	2 (22.2%)	5 (55.6%)	1 (11.1%)	8 (88.9%)	3 (33.3%)	6 (66.7%)	4 (0.8%)	4 (7.0%)	4 (7.0%)
Food Preparation and Serving Related: 4000–4160	13	1 (7.7%)	5 (38.5%)	5 (38.5%)	2 (15.4%)	2 (15.4%)	11 (84.6%)	0 (0%)	9 (69.2%)	4 (30.8%)	8 (61.5%)	5 (38.5%)	13 (2.7%)	0 (0%)	0 (0%)
Healthcare Pract. and Techs: 3000–3540	33	1 (3.0%)	2 (6.1%)	24 (72.7%)	6 (18.2%)	25 (75.8%)	7 (21.2%)	1 (3.0%)	33 (100%)	0 (0%)	33 (100%)	0 (0%)	32 (6.7%)	1 (1.8%)	1 (1.8%)
Healthcare Support: 3600–3655	11	0 (0%)	4 (36.4%)	7 (63.6%)	0 (0%)	8 (72.7%)	3 (27.3%)	0 (0%)	11 (100%)	0 (0%)	11 (100%)	0 (0%)	11 (2.3%)	0 (0%)	0 (0%)
Installation, Maintenance, and Repair: 7000–7630	37	0 (0%)	5 (13.5%)	8 (21.6%)	24 (64.9%)	3 (8.1%)	21 (56.8%)	13 (35.1%)	25 (67.6%)	12 (32.4%)	24 (64.9%)	13 (35.1%)	26 (5.5%)	11 (19.3%)	11 (19.3%)
Legal: 2100–2160	5	5 (100%)	0 (0%)	0 (0%)	0 (0%)	5 (100%)	0 (0%)	0 (0%)	5 (100%)	0 (0%)	5 (100%)	0 (0%)	5 (1.1%)	0 (0%)	0 (0%)
Life, Physical, and Social Science: 1600–1965	23	1 (4.3%)	5 (21.7%)	15 (65.2%)	2 (8.7%)	23 (100%)	0 (0%)	0 (0%)	20 (87.0%)	3 (13.0%)	20 (87.0%)	3 (13.0%)	23 (4.8%)	0 (0%)	0 (0%)
Management: 0010–0430	30	4 (13.3%)	4 (13.3%)	20 (66.7%)	2 (6.7%)	26 (86.7%)	4 (13.3%)	0 (0%)	26 (86.7%)	4 (13.3%)	25 (83.3%)	5 (16.7%)	30 (6.3%)	0 (0%)	0 (0%)
Material Moving: 9500–9750	14	0 (0%)	0 (0%)	2 (14.3%)	12 (85.7%)	2 (14.3%)	9 (64.3%)	3 (21.4%)	3 (21.4%)	11 (78.6%)	2 (14.3%)	12 (85.7%)	11 (2.3%)	3 (5.3%)	3 (5.3%)
Office and Administrative Support: 5000–5940	52	1 (1.9%)	45 (86.5%)	3 (5.8%)	3 (5.8%)	45 (86.5%)	5 (9.6%)	2 (3.8%)	49 (94.2%)	3 (5.8%)	49 (94.2%)	3 (5.8%)	52 (10.9%)	0 (0%)	0 (0%)
Personal Care and Service: 4300–4650	20	0 (0%)	3 (15.0%)	15 (75.0%)	2 (10.0%)	11 (55.0%)	7 (35.0%)	2 (10.0%)	19 (95.0%)	1 (5.0%)	19 (95.0%)	1 (5.0%)	20 (4.2%)	0 (0%)	0 (0%)
Production: 7700–8965	81	1 (1.2%)	6 (7.4%)	19 (23.5%)	55 (67.9%)	32 (39.5%)	39 (48.1%)	10 (12.3%)	78 (96.3%)	3 (3.7%)	55 (67.9%)	26 (32.1%)	73 (15.4%)	7 (12.3%)	7 (12.3%)
Protective Service: 3700–3955	18	2 (11.1%)	4 (22.2%)	8 (44.4%)	4 (22.2%)	4 (22.2%)	12 (66.7%)	2 (11.1%)	11 (61.1%)	7 (38.9%)	10 (55.6%)	8 (44.4%)	17 (3.6%)	1 (1.8%)	1 (1.8%)
Sales and Related: 4700–4965	18	0 (0%)	2 (11.1%)	16 (88.9%)	0 (0%)	17 (94.4%)	1 (5.6%)	0 (0%)	17 (94.4%)	1 (5.6%)	17 (94.4%)	1 (5.6%)	18 (3.8%)	0 (0%)	0 (0%)
Transportation: 9000–9420	22	0 (0%)	1 (4.5%)	8 (36.4%)	13 (59.1%)	12 (54.5%)	7 (31.8%)	3 (13.6%)	17 (77.3%)	5 (22.7%)	17 (77.3%)	5 (22.7%)	18 (3.8%)	3 (5.3%)	3 (5.3%)

Footer: Based on 2010 US Census Occupation Codes (COC); O*NET: Occupational Information Network; Meas: Measurement; Judg: Judgment; Construction: Construction and Extraction; Farming: Farming, Forestry, Fishing; Healthcare Pract. and Techs: Healthcare Practitioners and Technicians; Arts, Design, Entertainment, Sports, and Media: Arts/Entertainment, Sports, and Media.

**Table 2:** Proportion of California workforce by select occupation groups and race/ethnicity status within level of dimension using SARS-CoV-2/COVID job exposure matrix module.

		Talking Loudly (O*NET + Meas + Judgment)					Physical Activity (O*NET + Judg)				Cold (O*NET +Judg)			Hot (O*NET + Judg)		
		Not Loud	Loud	Some Loud	Very Loud	Total	Low	Medium	High	Total	Not Cold	Cold	Total	Hot	Not Hot	Total
Building, Grounds Cleaning and Maintenance: 4200–4250 (N = 665,161)	All	0	67.6	0	32.4	100	4.4	4.3	91.3	100	67.6	32.4	100	32.4	67.6	100
	AIAN	0	0	0	0.3	0.4	0	0	0.4	0.4	0	0.3	0.4	0.3	0	0.4
	Asian	0	3.4	0	0.4	3.7	0.1	0	3.7	3.7	3.4	0.4	3.7	0.4	3.4	3.7
	Black	0	2.2	0	0.4	2.5	0.3	0.2	2.1	2.5	2.2	0.4	2.5	0.4	2.2	2.5
	HPI	0	0.4	0	0	0.4	0	0	0.4	0.4	0.4	0	0.4	0	0.4	0.4
	Latino	0	49.3	0	25.9	75.2	2.4	2.7	70.1	75.2	49.3	25.9	75.2	25.9	49.3	75.2
	Multirace	0	0.6	0	0.1	0.7	0	0	0.7	0.7	0.6	0.1	0.7	0.1	0.6	0.7
	White	0	11.7	0	5.3	17.1	1.7	1.5	13.9	17.1	11.7	5.3	17.1	5.3	11.7	17.1
Computer and Mathematical: 1000–1240 (N = 803,604)	All	10.6	24.9	64.5	0	100	100	0	0	100	100	0	100	0	100	100
	AIAN	0	0	0.6	0	0.6	0.6	0	0	0.6	0.6	0	0.6	0	0.6	0.6
	Asian	2.9	7.9	33.9	0	44.7	44.7	0	0	44.7	44.7	0	44.7	0	44.7	44.7
	Black	1.2	1.4	1.5	0	4.1	4.1	0	0	4.1	4.1	0	4.1	0	4.1	4.1
	HPI	0.2	0.3	0.1	0	0.6	0.6	0	0	0.6	0.6	0	0.6	0	0.6	0.6
	Latino	1.8	4.2	4.4	0	10.5	10.5	0	0	10.5	10.5	0	10.5	0	10.5	10.5
	Multirace	0.2	0.4	1.4	0	2	2	0	0	2	2	0	2	0	2	2
	White	4.4	10.7	22.5	0	37.6	37.6	0	0	37.6	37.6	0	37.6	0	37.6	37.6
Construction: 6200–6940 (N = 950,742)	All	0	9.6	1.3	89	100	0	28	72	100	30.6	69.4	100	84.5	15.5	100
	AIAN	0	0	0	0.3	0.3	0	0.1	0.2	0.3	0.2	0.1	0.3	0.1	0.2	0.3
	Asian	0	0.5	0.1	2.2	2.8	0	1.4	1.4	2.8	1.1	1.7	2.8	2.5	0.3	2.8
	Black	0	0.5	0	2.6	3.1	0	1	2.1	3.1	0.7	2.4	3.1	2.8	0.3	3.1
	HPI	0	0.1	0	0.4	0.5	0	0.3	0.2	0.5	0.2	0.3	0.5	0.5	0	0.5
	Latino	0	4.9	0.7	58.8	64.4	0	14.1	50.3	64.4	18.9	45.5	64.4	53.1	11.2	64.4
	Multirace	0	0	0.2	1.1	1.3	0	0.7	0.7	1.3	0.4	1	1.3	1.3	0	1.3
	White	0	3.7	0.4	23.6	27.6	0	10.5	17.1	27.6	9.2	18.4	27.6	24.1	3.5	27.6
Farming: 6005–6130 (N = 243,348)	All	0	1.1	3.5	95.4	100	6.8	3.5	89.7	100	5.7	94.3	100	91.1	8.9	100
	AIAN	0	0	0	0.1	0.1	0	0	0.1	0.1	0	0.1	0.1	0.1	0	0.1
	Asian	0	0	0.1	3.2	3.3	0	0.1	3.2	3.3	0	3.3	3.3	3.3	0	3.3
	Black	0	0	0	0.3	0.3	0.3	0	0	0.3	0.3	0	0.3	0	0.3	0.3
	Latino	0	0.5	2.7	83.8	87	5.9	2.7	78.4	87	5.4	81.6	87	81.1	5.9	87
	Multirace	0	0	0.3	0	0.3	0	0.3	0	0.3	0	0.3	0.3	0.3	0	0.3
	White	0	0.6	0.4	7.9	8.9	0.6	0.4	7.9	8.9	0	8.9	8.9	6.2	2.7	8.9
Healthcare Pract. and Techs: 3000–3540 (N = 946,822)	All	1.7	40.6	8.4	49.2	100	49	48.2	2.8	100	100	0	100	0	100	100
	Asian	0.5	11.5	1.2	15.2	28.4	14.6	13.8	0.1	28.4	28.4	0	28.4	0	28.4	28.4
	Black	0.2	2.7	0.6	3.7	7.3	2.9	4.1	0.3	7.3	7.3	0	7.3	0	7.3	7.3
	HPI	0	0.4	0	0.4	0.8	0.2	0.6	0	0.8	0.8	0	0.8	0	0.8	0.8
	Latino	0.3	9	3.6	6	18.8	9.4	8.8	0.5	18.8	18.8	0	18.8	0	18.8	18.8
	Multirace	0	1.3	0.3	1.1	2.7	1.6	1.1	0	2.7	2.7	0	2.7	0	2.7	2.7
	White	0.7	15.7	2.8	22.8	42	20.2	19.9	1.9	42	42	0	42	0	42	42
Healthcare Support: 3600–3655 (N = 314,904)	All	0	57.3	42.7	0	100	52.3	47.7	0	100	100	0	100	0	100	100
	AIAN	0	0.9	0	0	0.9	0.5	0.4	0	0.9	0.9	0	0.9	0	0.9	0.9
	Asian	0	10.3	4.7	0	15	7.1	7.9	0	15	15	0	15	0	15	15
	Black	0	7.3	1.3	0	8.6	2	6.7	0	8.6	8.6	0	8.6	0	8.6	8.6
	HPI	0	1	0.8	0	1.8	0.8	1	0	1.8	1.8	0	1.8	0	1.8	1.8
	Latino	0	22.6	25.8	0	48.4	29.6	18.8	0	48.4	48.4	0	48.4	0	48.4	48.4
	Multirace	0	0.6	0.5	0	1.1	0.5	0.6	0	1.1	1.1	0	1.1	0	1.1	1.1
	White	0	14.5	9.6	0	24.2	11.9	12.3	0	24.2	24.2	0	24.2	0	24.2	24.2
Installation, Maintenance, and Repair: 7000–7630 (N = 467,393)	All	0	8.25	8.51	83.2	100	6.94	56.5	36.6	100	79.8	20.2	100	1.43	16.4	17.9
	AIAN	0	0	0.09	0.24	0.33	0	0.09	0.24	0.33	0.33	0	0.33	69.4	30.6	100
	Asian	0	0.92	1.34	8.72	11	1.55	7.1	2.32	11	9.5	1.48	11	0.13	1.61	1.74
	Black	0	0.09	0.72	2.86	3.67	0.22	1.75	1.7	3.67	3.24	0.43	3.67	2.87	3.36	6.23
	HPI	0	0.21	0.27	0.67	1.16	0.21	0.95	0	1.16	1.04	0.12	1.16	1.24	5.73	6.97
	Latino	0	2.93	4.23	42.6	49.8	2.28	28.6	18.9	49.8	40.2	9.61	49.8	0	0.99	0.99
	Multirace	0	0.28	0.09	0.98	1.35	0.28	0.61	0.45	1.35	1.26	0.09	1.35	15	49.9	64.8
	White	0	3.81	1.77	27.1	32.7	2.39	17.4	13	32.7	24.3	8.45	32.7	0	1.39	1.39
Material Moving: 9500–9750 (N = 479,414)	All	0	20.9	0	79.1	100	11	76	13	100	20.6	79.4	100	79.4	20.6	100
	AIAN	0	0.2	0	1.6	1.7	0.3	1.2	0.3	1.7	0.1	1.6	1.7	1.6	0.1	1.7
	Asian	0	2.6	0	3.6	6.2	0.4	5.4	0.5	6.2	2.9	3.4	6.2	3.4	2.9	6.2
	Black	0	1.5	0	5.4	7	0.8	6.1	0.1	7	1.2	5.7	7	5.7	1.2	7
	HPI	0	0	0	1	1	0.3	0.7	0	1	0	1	1	1	0	1
	Latino	0	14.6	0	50.3	64.8	7.9	47.2	9.8	64.8	15	49.9	64.8	49.9	15	64.8
	Multirace	0	0	0	1.4	1.4	0	1.4	0	1.4	0	1.4	1.4	1.4	0	1.4
	White	0	2	0	15.8	17.9	1.5	14	2.4	17.9	1.4	16.4	17.9	16.4	1.4	17.9
Production: 7700–8965 (N = 745,606)	All	0.2	31.7	7.4	60.7	100	46.5	26.4	27.2	100	84.4	15.6	100	30.3	69.7	100
	AIAN	0	0.2	0	0.3	0.5	0.1	0	0.4	0.5	0.3	0.2	0.5	0.2	0.3	0.5
	Asian	0	3.3	1	8.5	12.8	8.1	2.7	2.1	12.8	11.4	1.4	12.8	2.8	10	12.8
	Black	0	0.7	0	2.1	2.9	0.7	0.6	1.5	2.9	2.2	0.6	2.9	1	1.8	2.9
	HPI	0	0.4	0	0.2	0.6	0.3	0	0.3	0.6	0.3	0.3	0.6	0.4	0.2	0.6
	Latino	0	21.9	5.2	31.8	58.9	24.7	17.3	16.8	58.9	48.5	10.4	58.9	19.4	39.5	58.9
	Multirace	0	0	0.1	0.6	0.7	0.5	0.2	0	0.7	0.7	0	0.7	0	0.7	0.7
	White	0.1	5.2	1.1	17.3	23.7	12.2	5.5	6	23.7	21.1	2.6	23.7	6.4	17.3	23.7

Footer: Using proportions from Current Population Survey estimates for California, 2019; Based on 2010 US Census Occupation Codes (COC); O*NET: Occupational Information Network; Meas: Measurement; Judg: Judgment; Construction: Construction and Extraction; Farming: Farming, Forestry, Fishing; Healthcare Pract. and Techs: Healthcare Practitioners and Technicians; AIAN = American Indian/Alaska Native; HPI = Hawaiian and Pacific Islander.

## Data Availability

The SCoVJEM module may be requested for use by contacting the primary author.

## Supplementary Materials

The following supporting information can be downloaded at: https://www.mdpi.com/article/10.3390/ijerph22030448/s1. Table S1: A priori rubric and O*NET questions or criteria used to develop each dimension, SARS-CoV-2/COVID-19 job exposure matrix (SCoVJEM) module.

## Abbreviations

The following abbreviations are used in this manuscript:

AIAN	American Indian and Alaska Native
COC	U.S. Census Occupation Codes
HPI	Hawaiian and Pacific Islander
JEM	Job Exposure Matrix
Judg	Judgment
Meas	Measure
O*NET	Occupational Information Network
PA	Physical Activity
SCoVJEM	SARS-CoV-2 or COVID-19 Job Exposure Matrix
Se	Sensitivity
SOEM	SARS-CoV-2 Occupational Exposure Matrix
Sp	Specificity
