# Supplementary material for: Extending a COVID-19 Job Exposure Matrix: The SARS-CoV-2 or COVID-19 Job Exposure Matrix Module (SCoVJEM Module) for Population-Based Studies
Source: Int J Environ Res Public Health. 2025 Mar 18;22(3):448. doi: 10.3390/ijerph22030448 (PMC11942199; doi:10.3390/ijerph22030448)
Supplement: Supplementary file 1 [file ijerph-22-00448-s001.zip › ijerph-3429511-supplementary.pdf]

Table S1. *A Priori* rubric and O*NET questions or criteria used to develop each dimension, SARS-CoV-2/COVID-19 job exposure matrix (SCoVJEM) module

Questions		Talking Loudly	High Physical Activity	Cold Environments	Hot Environments
**A priori – Does the occupation involve?**	Q1	Working with loud machinery or equipment (power tools or turbines)	Moving multiple large muscle groups (e.g., running, playing sports)	The same indoor environment as products that require refrigeration (food, medical, industrial)	Environments with equipment or machines that generate heat in the work environment, such as: furnaces, smelter, boilers, ovens, steam-based generators, laundry, engines and other machines that generate heat
	Q2	Working in the music / performing / entertainment industry/stadiums or sporting events	Using of manual tools (e.g., digging, heavy constructions, pulling)	High altitude environments	Indoor environment without heating ventilation and air conditioning
	Q3	Working in environments related to transit/transportation (airports, trains)	Lifting or moving or handling heavy objects daily	High wind environments	Hot, outdoor environment during the day (i.e. summer)
	Q4	-	Involve climbing (e.g., stairs, poles, trees, ladders)	Cold, outdoor environment (i.e. winter)	Environments with geothermal heat exposure such as mines
	Q5	-	require walking and moving around more than half of the workday	Outside in the early morning hours or during the evening (no sun)	Environments with exposure to direct flame
	Q6	-	-	Lab or production areas with liquid nitrogen cooled equipment or chillers	-
	Q7	-	-	Wet environments e.g. marine equipment, fisheries	-
**O*NET Questions or Criteria**	Q1/C1	How often does this job require working exposed to sounds and noise levels that are distracting or uncomfortable? (Loud)	Performing physical activities that require considerable use of your arms and legs and moving your whole body, such as climbing, lifting, balancing, walking, stooping, and handling materials. (Phys Act)	How often does this job require working in very hot (above 90 F degrees) or very cold (below 32 F degrees) temperatures? (Extremes)	
	Q2/C2	How frequently does this job require the worker to deal with physical aggression of violent individuals? (Aggressive)	The ability to exert yourself physically over long periods of time without getting winded or out of breath. (Stamina)	-	-
	Q3/C3	How often do you have to have face-to-face discussions with individuals or teams in this job? (Face)	Spend time climbing ladders, scaffolds, or poles: How much does this job require climbing ladders, scaffolds, or poles? (Climb)	-	-
	Q4/C4		The ability to use short bursts of muscle force to propel oneself (as in jumping or sprinting), or to throw an object. (Explosive)		
